# Precision Fabrication and Optimization of Nanostructures for Exosome Detection via Surface-Enhanced Raman Spectroscopy

**DOI:** 10.3390/nano15040266

**Published:** 2025-02-10

**Authors:** Qingyi Wang, Bowen Yu, Bingbing Yang, Xuanhe Zhang, Guoxu Yu, Zeyu Wang, Hua Qin, Yuan Ma

**Affiliations:** 1 School of Mechanical-Electronic and Vehicle Engineering, Beijing University of Civil Engineering and Architecture, Beijing 102616, China; 2108550022075@stu.bucea.edu.cn (Q.W.); 2108550023065@stu.bucea.edu.cn (Z.W.); 2 Department of Mechanical Engineering, Tsinghua University, Beijing 100084, China; bowenyu@mail.tsinghua.edu.cn (B.Y.); xuanhe-z20@mails.tsinghua.edu.cn (X.Z.); ygx22@mails.tsinghua.edu.cn (G.Y.); 3 Department of Laboratory Medicine, Nanjing First Hospital, China Pharmaceutical University, Nanjing 210006, China; 3323092182@stu.cpu.edu.cn

**Keywords:** exosome detection, nanocone structure, SERS substrate

## Abstract

Exosome detection is crucial for biomedical research and clinical diagnostics due to their unique characteristics. Surface-enhanced Raman spectroscopy (SERS) based on nanostructure substrates with local field enhancement capability is a promising detection approach. However, the random distribution of nanostructures leads to uneven “hotspots” distribution, which limits their application in SERS detection. Here, we systematically investigated the impact of experimental parameters on nanostructure morphology and analyzed their formation mechanism, achieving controllable nanocone fabrication. Subsequent experiments confirmed the reliability and effectiveness of the fabricated nanocone in exosome SERS detection. This work not only realized flexible control of nanostructures but also expanded their application prospects in the field of exosome analysis.

## 1. Introduction

As nanoscale extracellular vesicles, exosomes are secreted by various cells, they play a critical role in intercellular communication, carrying biomolecules such as proteins, lipids, and nucleic acids [1,2,3]. Due to their correlation with various physiological and pathological processes, including cancer progression and immune response modulation, exosomes have emerged as valuable biomarkers for disease diagnosis and monitoring [4,5]. Precise detection and analysis of exosome are crucial for promoting biomedical research and clinical applications [6]. Over the past few decades, researchers have proposed various methods and techniques for exosome detection. While these studies have achieved some progress, they often suffer from limitations in sensitivity, specificity, and the ability to analyze molecular signatures comprehensively [7,8,9,10,11]. Among these methods, the surface-enhanced Raman scattering (SERS) emerged as a particularly promising approach. Compared with traditional methods, SERS has several outstanding advantages, including ultrahigh sensitivity up to the single-molecule level, molecular fingerprint recognition capabilities for precise biomolecular identification, and the potential for multiplex detection in complex biological samples [6,12,13,14]. For exosome detection, these attributes are especially important, as SERS can not only detect the presence of exosomes but also reveal their biological roles. Furthermore, SERS enables non-destructive analysis, preserving sample integrity for downstream applications [15,16,17]. These characteristics make SERS particularly well-suited for addressing the challenges of exosome detection, positioning it as a key focus of current research direction.

An ideal SERS substrate should possess strong electromagnetic field enhancement and effective localized surface plasmon resonance (LSPR) effects, which are intrinsically linked to the size, arrangement, and distribution of the nanoparticles (NPs) constituting the substrate [18,19,20,21]. Currently, to enhance localized field effects and improve detection sensitivity, nanostructures with small gaps have gained increasing attention [22]. Among these, nanocone structures stand out as a SERS substrate candidate due to their high aspect ratio and sharp tip, which exhibit stronger electromagnetic field enhancement effects, making them highly promising for SERS applications [12,23,24,25,26,27]. However, the random distribution of nanostructures can lead to uneven “hotspot” distribution, resulting in signal fluctuations and poor reproducibility in Raman spectral analysis [25,28,29,30]. Therefore, the functional effectiveness of the nanocone structures depend largely on aspects such as such as size control, shape optimization, arrangement patterns, as well as the feasibility and reliability of mass manufacturing. Common nanocone fabrication techniques include laser [31,32], nanoimprinting [33,34], metal-assisted chemical etching (MACE) [35,36], and deposition [37,38]. However, these methods often face challenges related to high cost, limited scalability, and precise morphology control.

**Figure 1:**
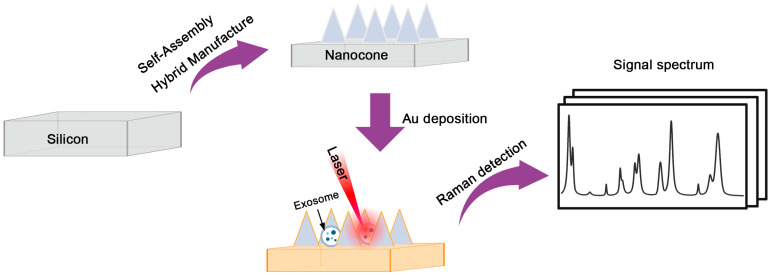
Schematic of the fabrication and application of nanocone structures for SERS detection, including nanocone fabrication, gold coating, and Raman signal measurement.

Herein, we systematically investigated the precise fabrication and optimization of silicon-based nanocone structures for exosome detection via Raman spectroscopy based on our proposed Self-Assembly Hybrid Manufacture technologies [39,40,41,42]. We investigated the control of nanostructure dimensions and morphology by varying experimental conditions, such as etching time and gas ratios. Additionally, we demonstrated the enhancement ability of the structure as the SERS substrate. Finally, the feasibility and reliability of the nanocone array we prepared as a promising substrate for exosome detection were verified. The schematic diagram is shown in Figure 1. The fabrication principles reported in this work realized flexibly control the arrangement and morphology of nanostructures by adjusting specific parameters, enabling the rapid and controllable fabrication of nanocone structures. Moreover, this work expands the potential applications of nanostructures in the field of biomedical detection. Future, nanostructures with different morphologies may be required as SERS substrates for detecting various analytes. Our study provides a valuable reference for promoting the development of SERS technology in subsequent applications.

## 2. Materials and Methods

### 2.1. Substrates Preparation

The silicon wafer was ultrasonically cleaned in acetone, ethanol, and deionized water for 5 min and then dried naturally. Next, the hydrophobic monodisperse colloidal solution of polystyrene nanoparticle (PS) (diameter 900 nm, 2.5% *w*/*v*, Baseline Chromtech Research Center, Tianjin, China) was added to the deionized water, and the hydrogel containing sodium dodecyl sulfate (SDS) was inserted into the water surface to make the PS densely stacked. The substrate was then removed from the solution to obtain a substrate assembled with PS mask. After the PS-coated silicon surface naturally dried, the samples were processed using the Inductively Coupled Plasma system (GSE-C200 RIE, NAURA, Beijing, China). The system was initially run through a predefined cleaning program to remove residual gases and ensure contamination-free processing. Next, the mask size was first shrunk in the O_2_ plasma environment to partially expose the substrate. Following this, in the same system, the specific gas combination SF_6_ and O_2_ was used to perform plasma etching on the PS nanoparticles and the silicon substrate, creating a highly uniform and ordered nanostructure array. The etching parameters, including the etching time and SF_6_/O_2_ ratio, all influence the final morphology of the nanostructure, which will be discussed in detail in the results Section 3. Finally, after the etching process, the silicon wafer was subjected to ultrasonic to remove the mask (Figure S1).

### 2.2. Cell Culture and Exosome Isolation

Human breast cancer cell line MCF-7 (Cell Resource Center, Peking Union Medical College, Beijing, China) was cultured in DMEM medium supplemented with 1% penicillin–streptomycin, 1% insulin, and 10% fetal bovine serum at 37 °C in a humidified incubator with 5% CO_2_. Once the cells reached approximately 90% confluency, the medium was replaced with a serum-free medium. After 24 h, the supernatant of the cell culture was collected. To remove cells and cellular debris, the collected supernatant was pretreated by centrifugation at 2000× *g* for 30 min at 4 °C. Exosomes were then extracted using a commercial exosome isolation kit (Thermo Fisher Scientific, Waltham, MA, USA) and stored at −80 °C for long-term preservation.

### 2.3. Raman Spectroscopy Detection

The fabricated nanocone structures were coated with a 90 nm Au film (with a 10 nm Ti adhesion layer) using electron-beam evaporation. Then, 10 μL of the prepared exosome solution was dropped onto the surface of the fabricated substrate. After natural drying, optical detection was performed using a Raman spectrometer (LabRAM HR Evolution, HORIBA, Kyoto, Japan) with a laser excitation wavelength of 785 nm. The measured Raman spectra were processed using LabSpec 6.3 software integrated with the measurement instrument.

### 2.4. Characterization

Using a high-resolution field emission scanning electron microscope (SEM) (Zeiss GeminiSEM, Oberkochen, Germany) with an accelerating voltage of 10 kV, we characterized the self-assembled and etched nanoparticles. The nanostructures were subsequently examined at appropriate tilt angles to assess their surface morphology. The SEM results were then processed using image analysis software such as ImageJ (v1.54k), which was employed to statistically summarize and post-process various data, including nanostructure height, top diameter, bottom diameter, etc.

## 3. Results and Discussion

### 3.1. Fabrication and Optimization Analysis of Nanocone Substrate

**Figure 2:**
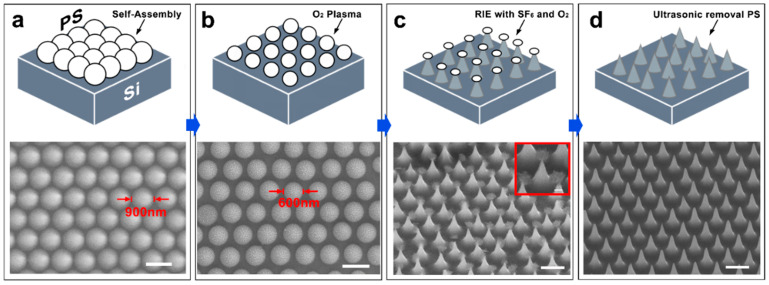
The schematic diagram (top) and representative SEM images (bottom) of the process for fabricating nanocone arrays. (**a**) The PS is transferred onto the silicon substrate through self-assembly. (**b**) O_2_ plasma treatment reduces the particle size of the mask. (**c**) The silicon substrate is etched into conical structures. The inset shows an image of a single nanocone structure (scale bar 200 nm). (**d**) Ultrasonic removal of the mask. The corresponding SEM images for each stage are shown below the schematic. Scale bar 1 μm.

**Figure 3:**
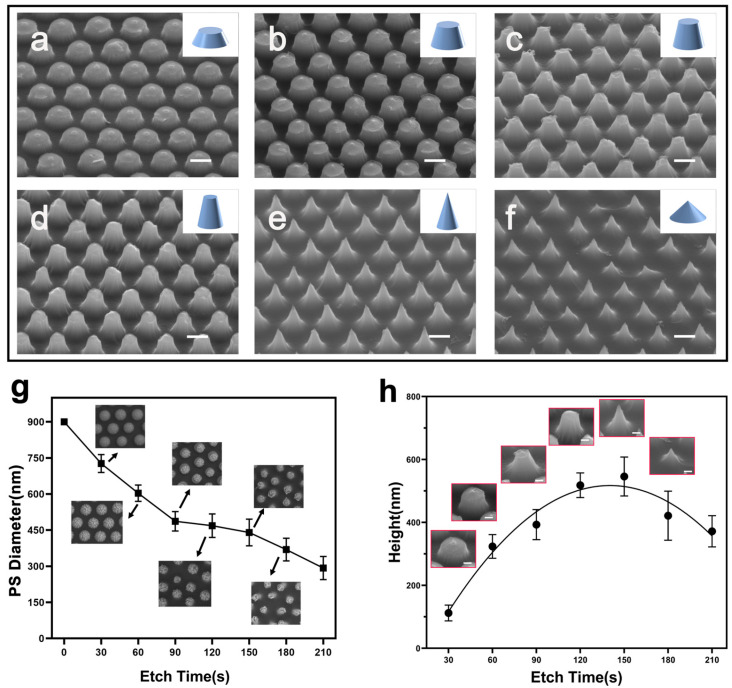
The representative SEM images show the evolution of the nanostructures and PS mask during the etching process. (**a**–**f**) SEM images at different etching times: (**a**) 30 s, (**b**) 60 s, (**c**) 90 s, (**d**) 120 s, (**e**) 150 s, and (**f**) 180 s, illustrating the morphological evolution of both the PS mask and nanostructures. Scale bar: 500 nm. (**g**) The PS mask diameter evolution as a function of etching time. The inset SEM images display representative top-view morphologies at each time point. (**h**) Average height statistics of the nanostructures at different etching times, with inset SEM images showing the corresponding morphologies. The scale bar in the insets is 200 nm.

We adopted the method mentioned in the previous study [42] to produce the nanocone structures. The entire process is illustrated in Figure 2. First, polystyrene (PS) colloidal nanoparticles were transferred onto a Si substrate using the self-assembly technique (Figure 2a) [39,40,43]. The O_2_ plasma etching was then applied to shrink the size of the PS particles, followed by RIE etching with an SF_6_/O_2_ gas mixture to shape the silicon into nanocone (Figure 2b,c) (details of the etching schematic and reactions are provided in Figure S2 and Figure 3). Finally, ultrasonic cleaning removed the residual mask, resulting in a well-aligned and highly uniform nanocone array (Figure 2d).

Firstly, etching time is one of the key factors determining the morphology of nanostructures throughout the experiment. Therefore, our initial focus was determining the optimal etching time for nanocone formation. Based on previous studies and practical working conditions, we set the initial flow rate of SF_6_ and O_2_ to 10 sccm and 15 sccm, respectively. Figure 3 shows the SEM images of the nanostructures at different time points after the mask was removed (Figure S4 shows the results with the mask).

The results indicate that both the mask and nanostructures exhibit significant changes at each time point. With increasing etching time, the role of the mask can be divided into two stages: active protection (I) and ineffective protection (II). In Stage I, the PS mask remains nearly spherical, effectively protecting the silicon substrate, leading to a relatively uniform etching process. The silicon surface gradually forms a cylindrical structure. As time increases into Stage II, the consumption of the PS mask accelerates, and its protective ability decreases. At this point, the top diameter of the nanostructures begins to shrink, forming conical structures with larger sidewall angles. Eventually, with prolonged etching, the PS mask significantly deteriorates, becoming flocculent and losing its protective function. The tip of nanostructures further reduces, forming needle-like structures. However, once the mask loses its protective ability, continued etching results in over-etching of the nanostructures, causing a reduction in the height of the nanocone. The corresponding results are shown in Figure 3a–f, which depicts the nanostructures after mask removal. We also characterized the nanostructures over a large area (Figure S5) to confirm the reliability and validity of the results. Additionally, the size of mask and height changes of nanostructures over time are shown in Figure 3g,h.

From the above results and measurements, we concluded that the etching time plays a critical role in determining the morphology of nanostructures by affecting the evolution of the PS mask and its protection ability. The mask will change from effective protection to ineffective protection. In addition, long-term etching after mask degradation will lead to over-etching and reduced nanocone height. These findings emphasize the importance of optimizing etching time to achieve the desired nanostructure morphology.

After determining the optimal etching time for nanocone formation, we further investigated the influence of gas flow rates on the resulting nanostructures. As critical components in the etching mechanism, SF_6_ and O_2_ play pivotal roles in determining the anisotropy and aspect ratio of the nanocone. Subsequent studies aim to elucidate the relationship between gas flow changes and nanostructure formation in order to optimize etching conditions.

**Figure 4:**
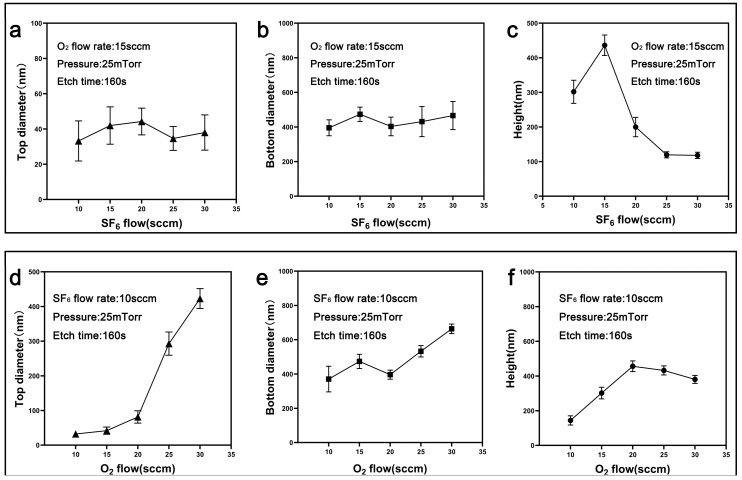
Effect of SF_6_ and O_2_ flow rates on nanostructure dimensions. (**a**–**c**) Top diameter, bottom diameter, and height as a function of SF_6_ flow rate at a fixed O_2_ flow rate of 15 sccm. (**d**–**f**) Top diameter, bottom diameter, and height as a function of O_2_ flow rate at a fixed SF_6_ flow rate of 10 sccm.

To analyze the effects of each gas individually, the etching time was fixed at 150 s while other experimental parameters remained constant. The SF_6_ flow rate was varied first, with the O_2_ flow rate held constant at 15 sccm. As shown in Figure S6, increasing the SF_6_ flow rates enhanced isotropic etching, resulting in a significant reduction in the aspect ratio. At higher SF_6_ flow rates, over-etching led to substrate damage and irregular surface morphologies. As shown in Figure 4a–c, the top and bottom diameters remained relatively stable across different SF_6_ flow rates, whereas the height exhibited a notable peak when the SF_6_ and O_2_ flow rates were balanced. Beyond this point, excessive SF_6_ flow significantly reduced the etching depth, leading to a sharp height decline.

Conversely, when the SF_6_ flow rate was fixed at 10 sccm and the O_2_ flow rate was increased, the nanostructures also exhibited distinct changes (Figure S6). This phenomenon indicates that while moderate O_2_ flow rates enhance anisotropic etching, excessive concentrations promote lateral etching, thereby hindering the vertical growth of the structures. Corresponding measurements in Figure 4d–f reveal that the top diameter consistently increased, while the bottom diameter displayed a non-monotonic trend, stabilizing at higher O_2_ flow rates. Similarly, the height initially increased with the O_2_ flow rate but decreased when the flow rate exceeded the optimal range.

**Figure 5:**
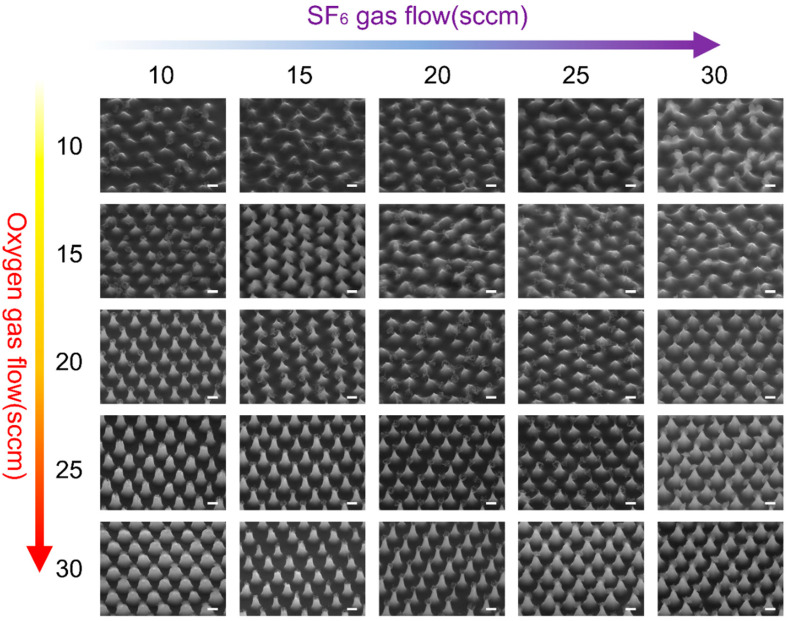
The SEM images show the morphological evolution of the mask and nanostructures etched under varying SF_6_ and O_2_ gas flow rates. The horizontal axis represents increasing SF_6_ gas flow (from 10 to 30 sccm), and the vertical axis represents increasing O_2_ gas flow (from 10 to 30 sccm). As the ratio of SF_6_ to O_2_ changes, distinct variations in anisotropy and aspect ratio are observed. Scale bar 500 nm.

These findings highlight the intricate balance between SF_6_ and O_2_ in controlling etching directionality and selectivity. Besides, they underscore the limitations of adjusting a single gas for precisely tailoring nanocone morphology, as neither SF_6_ nor O_2_ alone can achieve the desired combination of high aspect ratio and uniform structure. To address this issue, the combined effects of both gases were systematically studied by varying the SF_6_/O_2_ ratio (Figure 5). The results emphasize the importance of balancing SF_6_ and O_2_ interactions to optimize anisotropy and selectivity during the etching process. Notably, at certain gas ratios (e.g., SF_6_/O_2_ = 15:20 and 20:25), the nanostructures exhibited optimal morphologies, demonstrating the critical role of gas ratio control. By fine-tuning the SF_6_/O_2_ ratio, the etching direction and rate can be modulated, enabling precise adjustments to height and diameter to optimize nanocone formation.

### 3.2. Raman Performance Characterization of the Fabricated Substrate

From the above analysis of nanocone fabrication, we have demonstrated the ability to produce a variety of nanostructures with different morphologies. In this section, we aim to validate the functionality of these nanostructures.

To evaluate the Raman performance of the nanocone structures, we selected several well-shaped nanocone types fabricated using the aforementioned method and sputtered an Au layer onto their surfaces. We first calculated the enhancement factor (EF) using the standard dye molecule Rhodamine 6G (R6G) to evaluate the enhancement capability. By comparing the intensity of characteristic peaks from various nanocone structures and silicon substrate (with Au layer), we found that the Raman signal intensity detected by different types of nanocone for the dye molecule was generally similar. Therefore, we calculated the EF value based on the characteristic peak at 1360 cm⁻^1^ (aromatic C-C stretching vibration [44]), and obtained the EF value calculated to be ~1.8 × 10^5^, indicating that the nanocone structures provided notable enhancement ability, confirming their potential as effective SERS substrates. (detailed calculation was provided in Figure S8).

**Figure 6:**
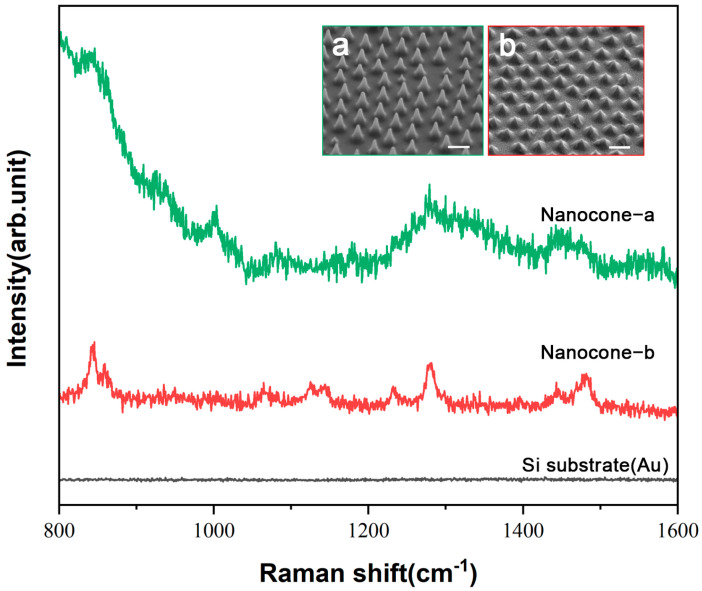
The raw Raman spectra of exosome on nanocone (**a**,**b**) and silicon substrate. The inset shows the nanostructure morphology and parameters, scale bar 1 μm. The exosome concentration is 10^9^ p/mL, and the laser wavelength is 785 nm.

Next, a solution of exosome prepared from MCF-7 cells (10^9^ particles/mL) was then deposited onto the nanostructures, followed by SERS measurements. We compared with those from the substrate (with Au layer) to evaluate the feasibility of this platform. The results indicate that the nanocone structures significantly enhanced the Raman signal of exosome molecules compared to the silicon substrate (Figure 6). However, significant differences were noted among the different nanocone structures. Specifically, structure ‘b’ exhibited more distinct characteristic peaks, while structure ‘a’ displayed stronger background signal, which could potentially obscure the characteristic exosome-specific signals. This indicates that the nanocone structures with characteristics like ‘b’ could be more suitable for enhancing signals from exosome.

**Figure 7:**
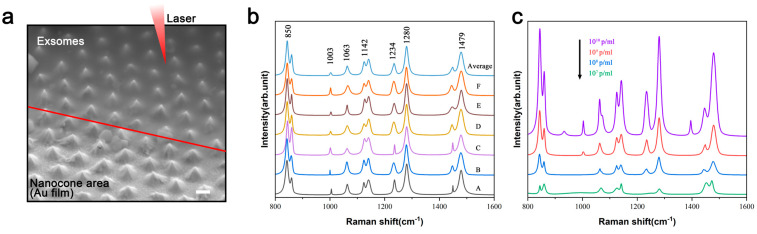
(**a**) The nanocone structure was pre-coated with an Au layer. The region above the red line represents the exosome area, where the laser is irradiated during Raman signal detection. Scale bar 1 μm. (**b**) Raman spectra and average spectra of exosome molecules (10^9^ p/mL) from six positions (A–F) on the nanocone structure. (**c**) Raman spectra of exosome molecules at different concentrations: from top to bottom, 10^10^ p/mL, 10^9^ p/mL, 10^8^ p/mL, and 10^7^ p/mL. The laser wavelength is 785 nm.

To verify this phenomenon, we repeated experiments on similar nanocone structure (Figure 7a). Raman spectra were collected from six random locations (A–F) within regions containing exosome, and the average spectra after processing are shown in Figure 7b. The results demonstrate that the nanocone structure effectively detected exosome signals with excellent signal uniformity across different locations. Additionally, the characteristic peaks of exosome were consistent with previous studies (the assignment of the main characteristic peaks is provided in Supplementary Table S1). This confirms the feasibility of the fabricated nanocone arrays as SERS substrates for exosome detection. To evaluate the reliability of the nanocone structures for exosome detection, additional measurements were performed using exosome solutions of varying concentrations, and the average spectra were compared (Figure 7c). The Raman spectra indicated a gradual decrease in signal intensity with decreasing exosome concentration. These findings suggest that our substrate is not only a promising candidate for detecting a wide range of biomolecules or chemicals but also has potential for quantitative applications, such as concentration calibration and analyte identification.

## 4. Conclusions

In conclusion, the nanocone arrays fabricated in this study have shown tremendous potential as effective substrates for Raman detection, particularly for exosome molecules. Although the Raman signal detection experiments are still in the preliminary stages, the results demonstrate that the nanocone structures significantly enhance the Raman signals from exosome, making them a promising and reliable detection platform. We believe that the ability to fine-tune the nanocone morphology can effectively enable Raman signal detection for a wide range of complex analytes, including exosomes and other microscopic substances. With further optimization of the nanocone arrangement and morphology, this platform also holds great potential for quantitative analysis, including concentration calibration and the identification of various analytes. This work could contribute to advancements in early disease diagnosis, environmental monitoring, and other fields requiring high-sensitivity detection in the future.

## Data Availability

Data are contained within the article or Supplementary Materials.

## Supplementary Materials

The following supporting information can be downloaded at: https://www.mdpi.com/article/10.3390/nano15040266/s1. Figure S1: (a) The photograph of the silicon wafer used in the experiment. (b) The optical image of the wafer surface with a PS nanoparticle monolayer assembled before etching. (c) The SEM image shows a PS nanoparticle monolayer arranged on a silicon substrate. (d) The optical image of the wafer surface after RIE etching. (e) The SEM image shows the microstructure produced after etching, indicating uniform nanoneedle formation. Scale bar 1 μm. Figure S2: Schematic of the formation process and reaction mechanism of nanocone etched on a silicon substrate with PS colloidal mask using SF_6_ and O_2_ gases in RIE etching. Figure S3: Simulation results of the time evolution of the mask and substrate during the etching process. (a,b) The mask gradually shrinks over time, while nanostructures begin to form on the substrate. (c,d) Structural changes after the mask is disregarded. Figure S4: The SEM images show the evolution of the PS mask and nanostructure over time during the etching process. The etching period is divided into two phases: (a–c) phase Ⅰ; (d,e) phase Ⅱ. Scale bar 500 nm. Figure S5: Low-magnification SEM images show the time-evolved morphology of the nanostructures with PS mask. The time is: (a) 30 s, (b) 60 s, (c) 90 s, (d) 120 s, (e) 150 s, (f) 180 s. These images illustrate the overall arrangement and uniformity of the nanostructures after etching via the SAHM method at different times, emphasizing the consistency and repeatability of the structures. Scale bar 1 μm. Figure S6: The SEM images show the effect of SF_6_ and O_2_ flow rates on nanostructure morphology. (a–e) Nanostructures etched with increasing SF_6_ flow rates(10–30 sccm), while keeping O_2_ flow rate constant at 15 sccm. (f–j) Nanostructures etched with increasing O_2_ flow rates(10–30 sccm), while keeping SF_6_ flow rate constant at 10 sccm. Scale bar 500 nm. Figure S7: (a) Schematic diagram of the FDTD simulation model, illustrating the nanocone array on the substrate and the incident light source. (b) Cross-sectional electric field distribution along the x-z plane, showing the field enhancement at the nanocone tip and gap. (c) Top-view electric field distribution on the x-y plane, highlighting the periodic field enhancement pattern across the nanocone array. Figure S8: The Raman spectra of R6G measured on the nanocone (red curve) and the silicon sunstrate (black curve); Table S1: SERS peak positions and tentative assignments of exosome. References [45,46,47,48,49,50,51,52,53,54,55,56,57,58,59,60,61,62,63,64,65] are cited in the supplementary materials.
