# Supplementary material for: Precision Fabrication and Optimization of Nanostructures for Exosome Detection via Surface-Enhanced Raman Spectroscopy
Source: Nanomaterials (Basel). 2025 Feb 10;15(4):266. doi: 10.3390/nano15040266 (PMC11858208; doi:10.3390/nano15040266)
Supplement: Supplementary file 1 [file nanomaterials-15-00266-s001.zip › Supporting information/Supporting information.pdf]

Supporting Information

Precision Fabrication and Optimization of Nanostructures for Exosome Detection via Raman Spectroscopy

Qingyi Wang[a], Bowen Yu[b], Bingbing Yang[c], Xuanhe Zhang[b], Guoxu Yu[b], Zeyu Wang[a], Hua Qin[a], Yuan Ma[b]**

[a] *School of Mechanical-Electronic and Vehicle Engineering, Beijing University of Civil Engineering and Architecture, Beijing 102616, P.R. China*

[b] *Department of Mechanical Engineering, Tsinghua University, Beijing 100084, P.R. China*

[c] *Department of Laboratory Medicine, Nanjing First Hospital, China Pharmaceutical University, Nanjing 210006, China*

**Yuan Ma - Email: yuanma@tsinghua.edu.cn*

**Hua Qin - Email: qinhua@bucea.edu.cn*

S1. Surface Changes of the Substrate Before and After Processing.

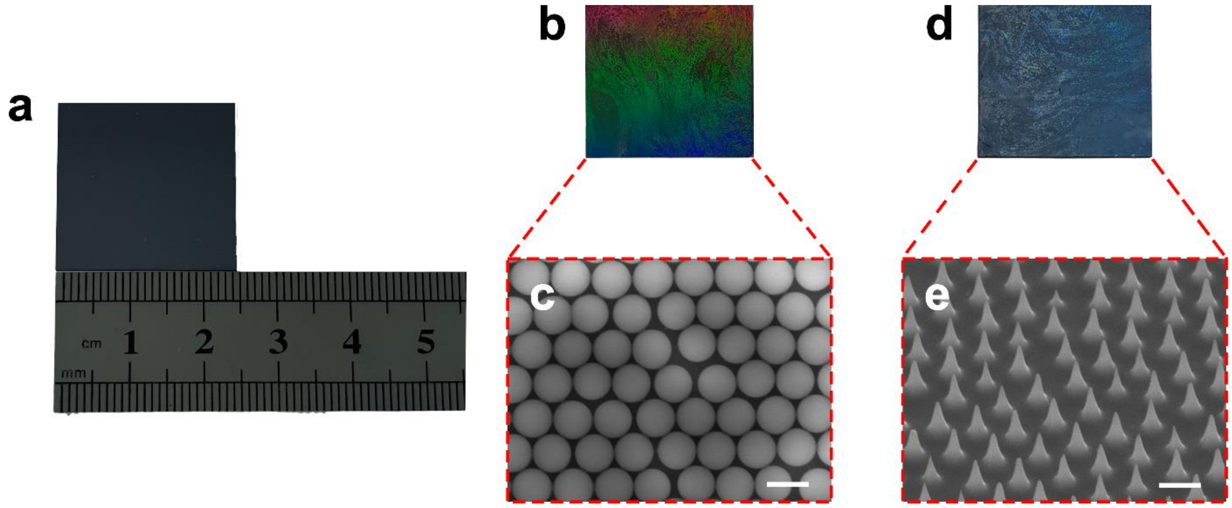

Figure S1. (a) The photograph of the silicon wafer used in the experiment. (b) The optical image of the wafer surface with a PS nanoparticle monolayer assembled before etching. (c) The SEM image shows a PS nanoparticle monolayer arranged on a silicon substrate. (d) The optical image of the wafer surface after RIE etching. (e) The SEM image shows the microstructure produced after etching, indicating uniform nanoneedle formation. Scale bar 1 μm.

S2. RIE Etching reaction and Simulation.

We reviewed relevant studies and conducted a detailed analysis of the SAHM method we employed. First, regarding RIE, this technique employs radio frequency (RF) to convert reactive gases into active species (ions or radicals, represented as '*'), which then interact with the target material through chemical and physical reactions to etch the desired nanostructures.[1-3] Fluorine-based gases like SF_6 and CF_4 are commonly used for their high etching rates and isotropic reactions with silicon.[4, 5] By introducing protective gases such as O_2, the overall etching rate and anisotropic profile control of the etching process can be improved.[6-8] The reactions involved are represented by the following equations:[1, 9-11]

$$e^- + SF_6 \rightarrow SF_x^* + F^* + e^- \ (x = 1 - 5) \quad (1)$$

$$e^- + O_2 \rightarrow 2O^* \quad (2)$$

$$Si + O^* + F^* \rightarrow SiO_xF_y (x = 1 - 2 \,; y = 4 - 5) \quad (3)$$

$$Si + 4F^* \rightarrow SiF_4 \uparrow \quad (4)$$

$$SF_4^* + SiO_xF_y \rightarrow SiF_4 \uparrow + SO_xF_y \uparrow (x = 1 - 2 \,; y = 4 - 5) \quad (5)$$

And the schematic diagram of the entire reaction process is shown in **Figure S2**. In the RF field, SF_6 and O_2 dissociate into SF_x*, F*, and O* radicals (Equations (1) and (2)). F* reacts with silicon, forming SiF_4 and causing isotropic etching. Low O_2 concentrations increase F* production, accelerating etching. The silicon surface also forms a SiO_xF_y passivation layer with F* and O*, preventing excessive etching and enhancing anisotropy (Equation (3)). Higher O_2 concentrations intensify this passivation, limiting vertical etching and nanostructure growth. SF_x can remove the passivation layer (Equation (5)), exposing silicon and continuing the etching cycle. The SF_6/O_2 ratio controls the free radicals and passivation layer dynamics, determining the final nanostructure morphology.

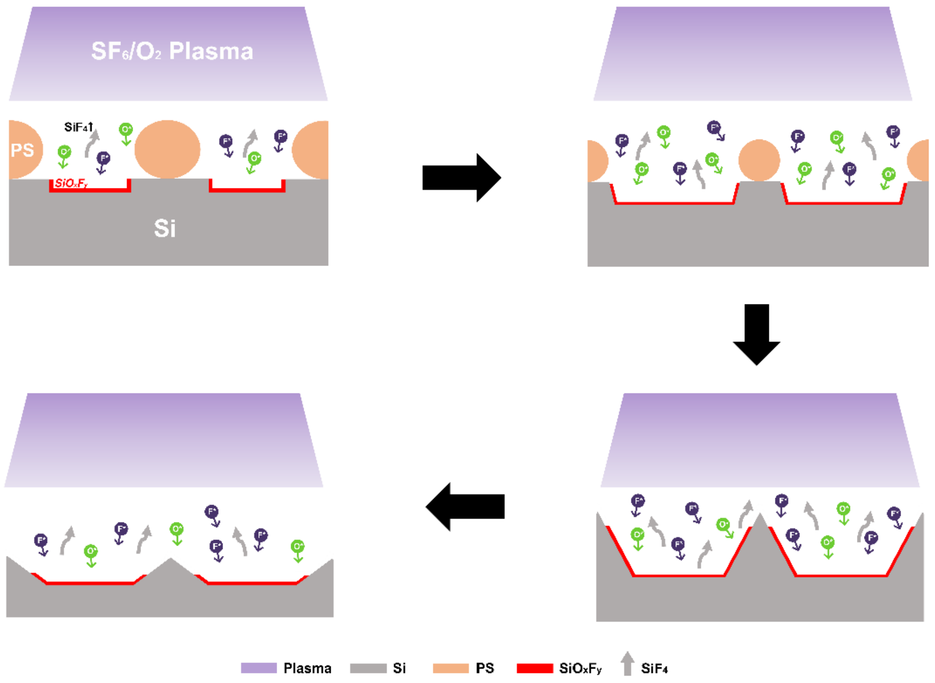

Figure S2. Schematic of the formation process and reaction mechanism of nanocone etched on a silicon substrate with PS colloidal mask using SF_6 and O_2 gases in RIE etching.

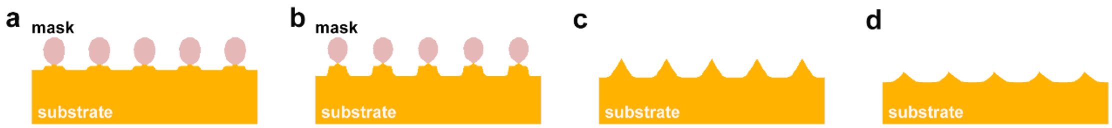

Figure S3. Simulation results of the time evolution of the mask and substrate during the etching process. (a, b) The mask gradually shrinks over time, while nanostructures begin to form on the substrate. (c, d) Structural changes after the mask is disregarded.

S3. Evolution of the Mask Morphology During Etching.

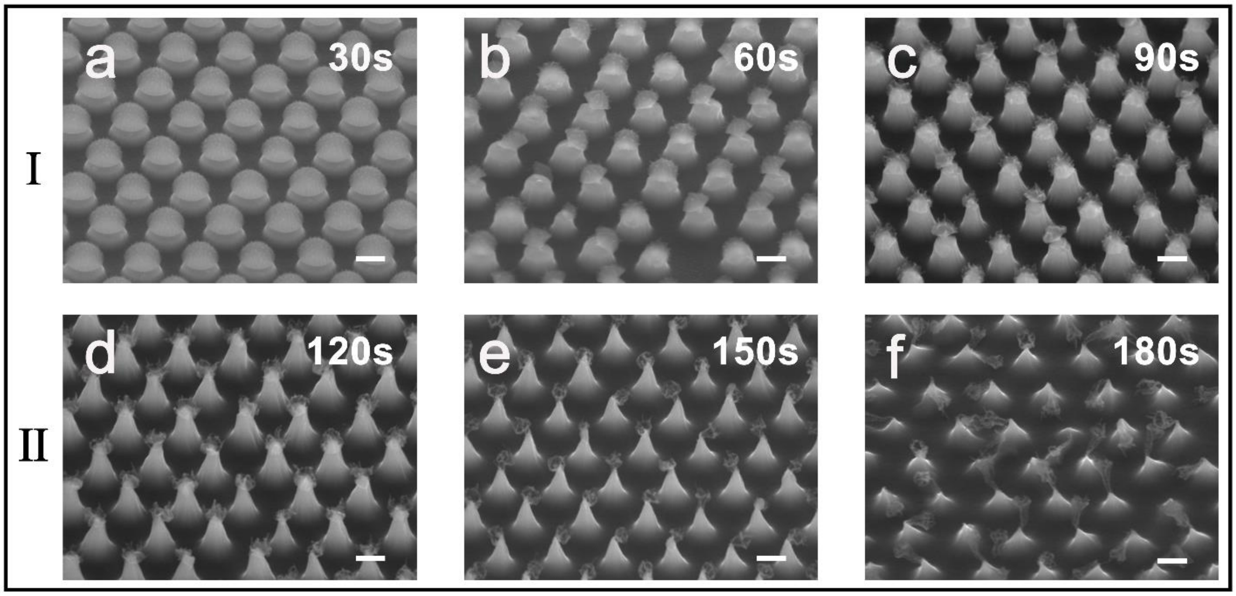

Figure S4. The SEM images show the evolution of the PS mask and nanostructure over time during the etching process. The etching period is divided into two phases: (a-c) phase I; (d-e) phase II. Scale bar 500nm.

S4. Characterization of Nanostructure Uniformity.

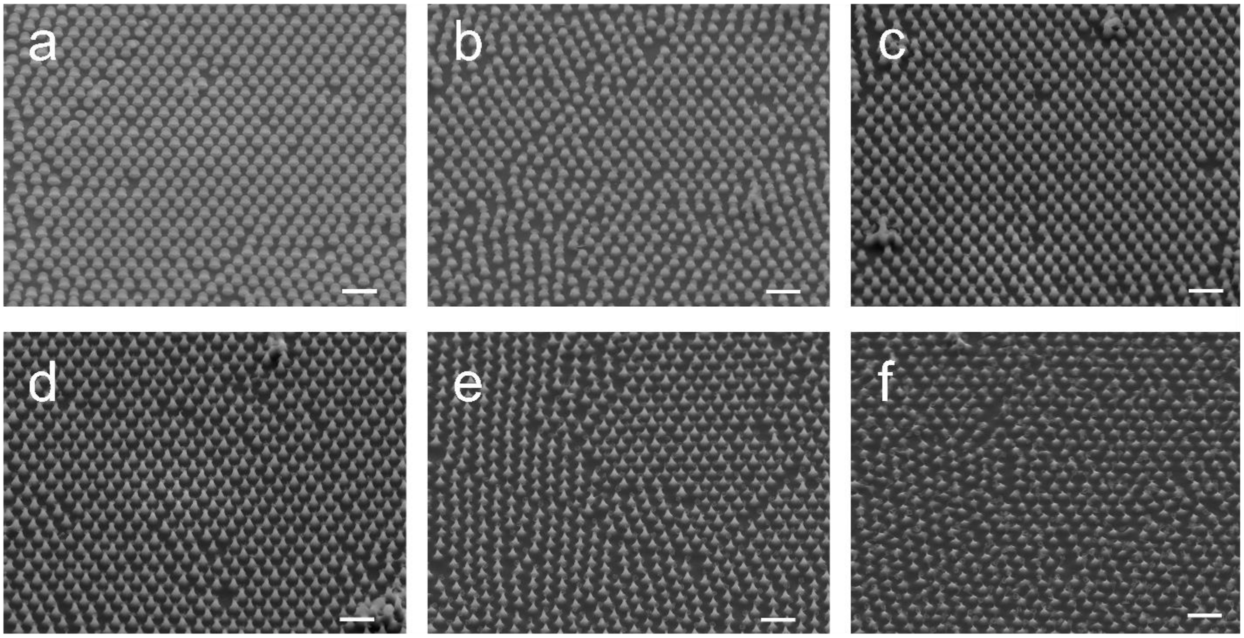

Figure S5. Low-magnification SEM images show the time-evolved morphology of the nanostructures with PS mask. The time is: (a)30s, (b)60s, (c)90s, (d)120s, (e)150s, (f)180s. These images illustrate the overall arrangement and uniformity of the nanostructures after etching via the SAHM method at different times, emphasizing the consistency and repeatability of the structures. Scale bar 1μm.

S5. Morphology changes with SF_6/O_2.

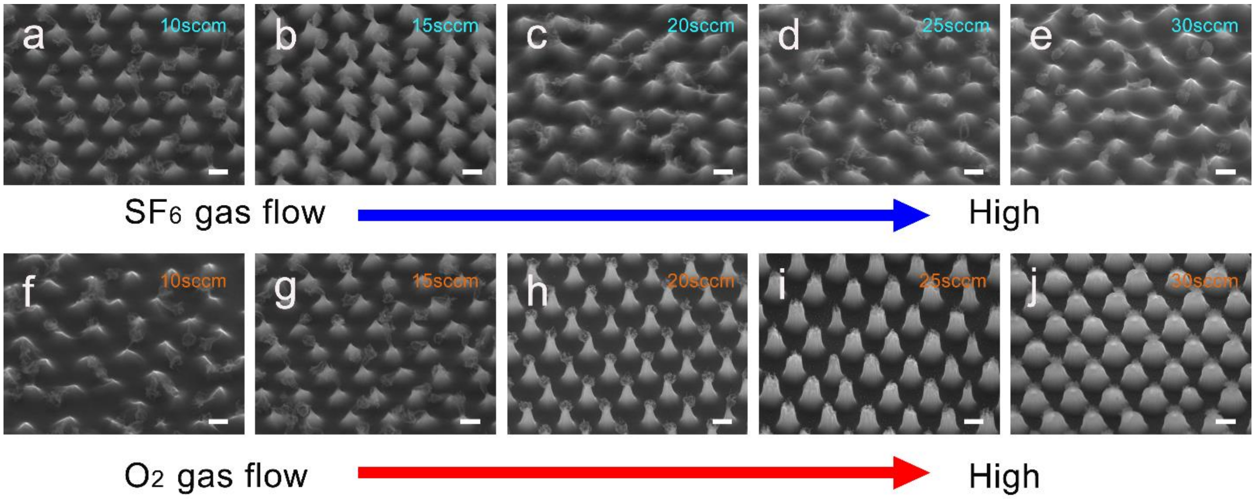

Figure S6. The SEM images show the effect of SF_6 and O_2 flow rates on nanostructure

morphology. (a-e) Nanostructures etched with increasing SF_6 flow rates(10-30sccm), while keeping O_2 flow rate constant at 15sccm. (f-j) Nanostructures etched with increasing O_2 flow rates(10-30sccm), while keeping SF_6 flow rate constant at 10sccm. Scale bar 500nm.

S6. Finite-Difference Time-Domain Simulation.

To better understand the localized surface plasmon resonance (LSPR) and surface-enhanced Raman scattering (SERS) effects of the fabricated nanocone structure, finite-difference time-domain (FDTD) simulations were performed. The simulation model was designed based on the geometry of the nanocone arrays, as illustrated in **Figure S7**a. The model consists of a rectangular unit cell with four uniform nanocones arranged in a regular pattern. Periodic boundary conditions were applied in two dimensions to simulate an infinite array, while perfect matching layers (PML) were used at the top and bottom to represent air and the gold-coated material. An automatic non-uniform mesh was employed to enhance numerical accuracy, and field monitors were positioned in the XZ and XY planes to capture electromagnetic field distributions. The simulations, performed using Lumerical software, included key steps such as material parameter definition, light source configuration, and mesh optimization. A plane wave with a wavelength of 785 nm and an electric field amplitude of 1 was used, with optical parameters for Au and Si derived from the Palik handbook.

As shown in Figure S7, the simulation results reveal significant field enhancement at the nanocone tips and gaps in the XZ plane (**Figure S7**b), consistent with the expected "hot spots." The XY plane (**Figure S7**c) shows a periodic enhancement pattern corresponding to the nanocone array. These results confirm the strong localized electromagnetic fields generated by the nanocone, supporting their potential as the SERS substrate. However, these simulations represent idealized conditions, and experimental validation is necessary to confirm the true enhancement caoabilities.

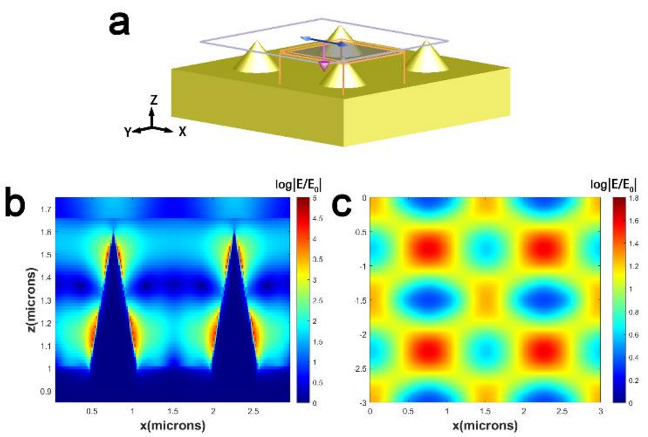

Figure S7. (a) Schematic diagram of the FDTD simulation model, illustrating the nanocone array on the substrate and the incident light source. (b) Cross-sectional electric field distribution along the x-z plane, showing the field enhancement at the nanocone tip and gap. (c) Top-view electric field distribution on the x-y plane, highlighting the periodic field enhancement pattern across the nanocone array.

S7. Calculation of Enhancement Factor (EF)

The general method for calculating the enhancement factor uses the following formula:

$$EF = \frac{I_{SERS}/N_{SERS}}{I_R/N_R} \tag{6}$$

where I_{SERS} and I_R represent the Raman intensities of a characteristic peak on the nanocone substrate and the normal substrate, respectively. N_{SERS} and N_R denote the number of molecules contributing to the Raman signal on the nanocone substrate and the normal substrate, respectively. The Rhodamine 6G(R6G) was used as the probe molecule, with concentrations of 10^{-6} M for the nanocone substrate and 10^{-2} M for the silicon substrate. To ensure uniform molecular coverage, both substrates with distinct surface areas were immersed in their respective dye solutions. The Raman spectra obtained from the two substrates (shown in **Figure S8**) were analyzed, with the signal intensity at 1360 cm^{-1} (aromatic C-C stretching vibration) chosen for EF calculation. The measured values were I_{SERS} = 5680 counts and I_R = 850 counts. By substituting these values into formula and correcting for the differences in molecular contributions based on the geometric properties of the substrates, the calculated EF was ~1.8×10^5, demonstrating the superior enhancement performance of the nanocone substrate.

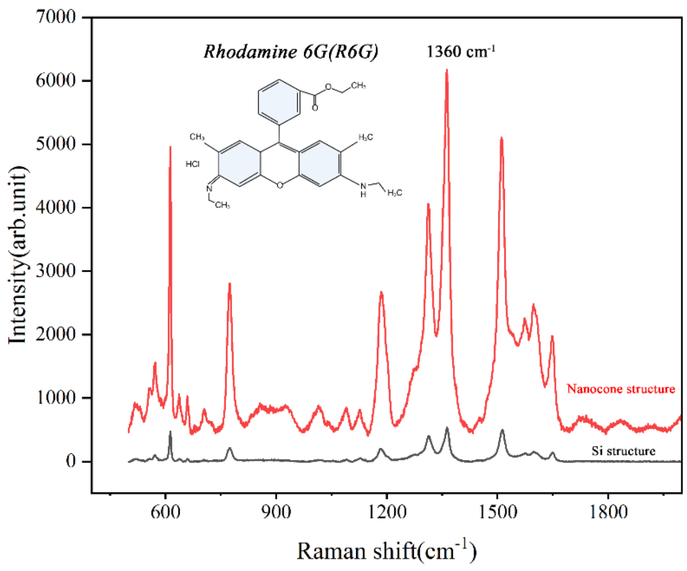

Figure S8. The Raman spectra of R6G measured on the nanocone (red curve) and the silicon sunstrate (black curve).

Table S1. SERS peak positions and tentative assignments of exosome. [12-21]

Raman shift (cm^{-1})	Peak assignment
855	C-C stretch proline ring in collagen
1003	Phenylalanine ring respiratory vibration
1063	C-C vibrations in lipid and protein
1142	CH vibration in protein
1234	Amide III (C-N, N-H bending)
1280	Amide III, C-H deformation
1479	C-N stretching, C-H deformation (e.g., lipid, protein)

References

1. Wakawaiachi, S. M.; Tezani, L. L.; Pessoa, R. S.; Medeiros, H. S.; Maciel, H. S.; Petraconi, G., Morphological and Chemical Analysis of Silicon Etched by SF6+O2 and CF4+O2 Low Pressure Constricted Plasma Jet. *ECS Transactions* **2011,** *39* (1), 409.
2. Alam, A. B. M. K.; Kuittinen, M.; Laukkanen, J. In *Etching Process Development of SiO 2 Etching Using Inductively Coupled Plasma*, 2015.
3. Tian, F.; Li, M.; Wu, S.; Li, L.; Hu, H., A hybrid and scalable nanofabrication approach for bio-inspired bactericidal silicon nanospike surfaces. *Colloids and Surfaces B: Biointerfaces* **2023,** *222*, 113092.
4. Arana, L. R.; de Mas, N.; Schmidt, R. T.; Franz, A. J.; Schmidt, M. A.; Jensen, K. F., Isotropic etching of silicon in fluorine gas for MEMS micromachining. *Journal of Micromechanics and Microengineering* **2007,** *17*, 384 - 392.
5. Pessoa, R. S.; Maciel, H. S.; Petraconi, G.; Massi, M.; da Silva Sobrinho, A. S., Effect of gas residence time on the morphology of silicon surface etched in SF6 plasmas. *Applied Surface Science* **2008,** *255* (3), 749-751.
6. Wongwanitwattana, C.; Shah, V. A.; Myronov, M.; Parker, E. H. C.; Whall, T.; Leadley, D. R., Precision plasma etching of Si, Ge, and Ge:P by SF6 with added O2. *Journal of Vacuum Science & Technology A* **2014,** *32* (3).
7. Aydinoglu, F.; Pan, A.; Zhu, C.; Cui, B., Effect of oxygen plasma cleaning on nonswitching pseudo-Bosch etching of high aspect ratio silicon pillars. *Journal of Vacuum Science & Technology B* **2020,** *38*, 012804.
8. Legtenberg, R.; Jansen, H. V.; Boer, M. D.; Elwenspoek, M. In *Anisotrapic Reactive Ion Etching of Silicon Using SF 6 / O 2 / CHF 3 Gas Mixtures*, 2005.
9. Chen, Y.; Xu, Z.; Gartia, M. R.; Whitlock, D.; Lian, Y.; Liu, G. L., Ultrahigh Throughput Silicon Nanomanufacturing by Simultaneous Reactive Ion Synthesis and Etching. *ACS Nano* **2011,** *5* (10), 8002-8012.
10. Zhiting, G.; Zhuang, M.; Lihong, G.; Qiang, L.; Yuxiang, W.; Yanbo, L.; Lidong, W.; Yuyang, H.; Yuanhan, D., Etching mechanism of high-aspect-ratio array structure. *Microelectronic Engineering* **2023,** *279*, 112060.
11. Chang, L.; Liu, X.; Luo, J.; Lee, C.-Y.; Zhang, J.; Fan, X.; Zhang, W., Physiochemical Coupled Dynamic Nanosphere Lithography Enabling Multiple Metastructures from Single Mask. *Advanced Materials* **2024,** *36* (13), 2310469.
12. Manciu, F. S.; Ciubuc, J. D.; Parra, K.; Manciu, M.; Bennet, K. E.; Valenzuela, P.; Sundin, E. M.; Durrer, W. G.; Reza, L.; Francia, G., Label-Free Raman Imaging to Monitor Breast Tumor Signatures. *Technology in Cancer Research & Treatment* **2017,** *16* (4), 461-469.
13. Zhang, P.; Wang, L.; Fang, Y.; Zheng, D.; Lin, T.; Wang, H., Label-Free Exosomal Detection and Classification in Rapid Discriminating Different Cancer Types Based on Specific Raman Phenotypes and Multivariate Statistical Analysis. *Molecules* **2019,** *24* (16), 2947.
14. Kallaway, C.; Almond, L. M.; Barr, H.; Wood, J.; Hutchings, J.; Kendall, C.; Stone, N., Advances in the clinical application of Raman spectroscopy for cancer diagnostics. *Photodiagnosis and Photodynamic Therapy* **2013,** *10* (3), 207-219.
15. Galler, K.; Requardt, R. P.; Glaser, U.; Markwart, R.; Bocklitz, T.; Bauer, M.; Popp, J.; Neugebauer, U., Single cell analysis in native tissue: Quantification of the retinoid content of hepatic stellate cells. *Scientific Reports* **2016,** *6* (1), 24155.
16. Willets, K. A., Surface-enhanced Raman scattering (SERS) for probing internal cellular structure and dynamics. *Analytical and Bioanalytical Chemistry* **2009,** *394* (1), 85-94.
17. Shetty, G.; Kendall, C.; Shepherd, N.; Stone, N.; Barr, H., Raman spectroscopy: elucidation of biochemical changes in carcinogenesis of oesophagus. *British Journal of Cancer* **2006,** *94* (10), 1460-1464.
18. Tirinato, L.; Gentile, F.; Di Mascolo, D.; Coluccio, M. L.; Das, G.; Liberale, C.; Pullano, S. A.; Perozziello, G.; Francardi, M.; Accardo, A.; De Angelis, F.; Candeloro, P.; Di Fabrizio, E., SERS analysis on exosomes using super-hydrophobic surfaces. *Microelectronic Engineering* **2012,** *97*, 337-340.
19. Kast, R. E.; Serhatkulu, G. K.; Cao, A.; Pandya, A. K.; Dai, H.; Thakur, J. S.; Naik, V. M.; Naik, R.; Klein, M. D.; Auner, G. W.; Rabah, R., Raman spectroscopy can differentiate malignant tumors from normal breast tissue and detect early neoplastic changes in a mouse model. *Biopolymers* **2008,** *89* (3), 235-241.
20. Diao, X.; Li, X.; Hou, S.; Li, H.; Qi, G.; Jin, Y., Machine Learning-Based Label-Free SERS Profiling of Exosomes for Accurate Fuzzy Diagnosis of Cancer and Dynamic Monitoring of Drug Therapeutic Processes. *Analytical Chemistry* **2023,** *95* (19), 7552-7559.
21. Xie, Y.; Su, X.; Wen, Y.; Zheng, C.; Li, M., Artificial Intelligent Label-Free SERS Profiling of Serum Exosomes for Breast Cancer Diagnosis and Postoperative Assessment. *Nano Letters* **2022,** *22* (19), 7910-7918.
